# Original article: role of adjuvant chemotherapy in a perioperative chemotherapy regimen for gastric cancer

**DOI:** 10.1186/s12885-016-2708-0

**Published:** 2016-08-18

**Authors:** Sven Lichthardt, Alexander Kerscher, Ulrich A. Dietz, Christian Jurowich, Volker Kunzmann, Burkhard H. A. von Rahden, Christoph-Thomas Germer, Armin Wiegering

**Affiliations:** 1 Department of General, Visceral, Vascular and Pediatric Surgery, University Hospital, University of Wuerzburg, Oberduerrbacherstr.6, 97080 Wuerzburg, Germany; 2 Comprehensive Cancer Centre Mainfranken, University Hospital, University of Wuerzburg, Josef-Schneiderstr. 6, 97080 Wuerzburg, Germany; 3 Department of Biochemistry and Molecular Biology, University of Wuerzburg, Am Hubland, 97074 Wuerzburg, Germany; 4 Department of Internal Medicine II, University of Wuerzburg Medical Center, Oberduerrbacherstr.2, 97080 Wuerzburg, Germany

**Keywords:** Gastric cancer, Chemotherapy, Adjuvant, Neoadjuvant, Multimodal, Risk factor, Complication, Survival

## Abstract

**Background:**

Multimodal treatment strategies – perioperative chemotherapy (CTx) and radical surgery – are currently accepted as treatment standard for locally advanced gastric cancer. However, the role of adjuvant postoperative CTx (postCTx) in addition to neoadjuvant preoperative CTx (preCTx) in this setting remains controversial.

**Methods:**

Between 4/2006 and 12/2013, 116 patients with locally advanced gastric cancer were treated with preCTx. 72 patients (62 %), in whom complete tumor resection (R0, subtotal/total gastrectomy with D2-lymphadenectomy) was achieved, were divided into two groups, one of which receiving adjuvant therapy (*n* = 52) and one without (*n* = 20). These groups were analyzed with regard to survival and exclusion criteria for adjuvant therapy.

**Results:**

Postoperative complications, as well as their severity grade, did not correlate with fewer postCTx cycles administered (*p* = n.s.). Long-term survival was shorter in patients receiving postCTx in comparison to patients without postCTx, but did not show statistical significance. In per protocol analysis by excluding two patients with perioperative death, a shorter 3-year survival rate was observed in patients receiving postCTx compared to patients without postCTx (3-year survival: 71.2 % postCTx group vs. 90.0 % non-postCTx group; *p* = 0.038).

**Conclusion:**

These results appear contradicting to the anticipated outcome. While speculative, they question the value of post-CTx. Prospectively randomized studies are needed to elucidate the role of postCTx.

## Background

Gastric cancer (GC) is the second most common cancer of the gastrointestinal tract, accounting for 6.8 % of all cancer diagnoses and 8.8 % of all cancer-related deaths. In 2012, there were 951,000 new cases of GC and 723,000 deaths due to GC worldwide [1–5]. In Japan and Korea the survival rate of patients with GC has increased over the past decade which may partially result from increased detection rates of early stage cancer due to screening programs in this area. Nevertheless, the overall 5-year survival rate in the western world remains low with apparently 30 %, less than 50 % for stage II cancer and lower than 20 % for stage III cancer [6, 7].

Surgical resection is the only potentially curative treatment for GC. To improve the poor outcome rate many studies have examined various aspects of surgical techniques, including extended lymph node dissection, the addition of perioperative or intraoperative radiotherapy and the effect of neoadjuvant (preCTx) as well as adjuvant (postCTx) chemotherapy and radiochemotherapy [8–19]. Conflicting results have been published for adjuvant chemotherapy. Compared to surgery alone, a survival advantage has been demonstrated in patients with adjuvant chemotherapy in Asian trials, while western studies have failed to reproduce this survival benefit [8–16]. On the other hand, in European trials, it has been shown that the application of neoadjuvant chemotherapy lead to significantly smaller tumors, less lymph node metastases, improved curative resection and improved overall and progression free survival compared to surgery alone [17–19]. Even so, this trails had several limitations, such as the inclusion of early stage GC, differences in lymphadenectomy and a low adjuvant CTx-rate.

### Study aims

We aimed to analyze the role of additional adjuvant chemotherapy (postCTx) in patients after preoperative CTx (preCTx) and curative radical surgery for locally advanced gastric cancer. We compared groups with postCTx and without postCTX regarding survival rate and analyzed exclusion criteria for post CTx.

## Methods

### Patient population

Data of patients having undergone preCTx and subsequent radical surgical resection for GC at the University Hospital of Wuerzburg, Germany (Universitätsklinik Würzburg, UKW) between January 1992 and December 2013 were retrieved from the Wuerzburg Institutional Database (WID). Patients were grouped according to the application of postCTx.

### Data source

The WID is a central prospective database, which has been expanded on a daily basis since 1984 with clinical, operative and research data of patients, who were evaluated and treated at the UKW. The collection of data and the scientific analysis are approved by an institutional review board. The UKW is one of three institutions treating patients with GC in an area with a population of about 515,000. Data available within the WID include patient demographics, histological diagnoses based on International Classification of Diseases coding standards, physician data, inpatient admission and outpatient registration data, operative procedures, laboratory values and computerized medication records. Continuous cross platform integration with the Wuerzburg Comprehensive Cancer Registry ensures updated follow-up information for the identification of deceased patients. Inpatient and outpatient records of all identified patients were reviewed retrospectively regarding type and duration of chemotherapy, sites of metastatic disease at presentation and disease status at last follow-up. Missing data was retrieved from patient records when possible.

Demographic details, clinical variables recorded at the time of primary diagnosis as well as during the initial operation (tumor site and the presence of any metastases) and histological details of the resected specimen (tumor (T) stage, nodal (N) stage, tumor differentiation (G) and evidence of microscopic venous (V) and lymphatic vessel invasion (L)) were compiled. This data was correlated with survival data obtained from prospective follow-up.

### Treatment

All patients presented with histologically proven GC at the hospital were staged by CT-scan of thorax and abdomen for distant metastases. Local staging was performed by endoscopic ultrasound. All patients underwent gastric resection with D2-lymphadenectomy. All patients were discussed in a multidisciplinary team conference at the time of diagnosis, after preCTx and after the operation.

### Follow-up

Postoperative follow-up consisted of quarterly outpatient assessments or the gathering of complete information from patients’ primary care physicians in 3-month intervals for at least 10 years. Follow-up was performed based on protocols according to entity and tumor stage with abdominal ultrasound after 3, 6, 12 and 18 months, followed by a yearly basis.

### Postoperative complication

Postoperative complications were classified according to the Dindo classification [20].

### Ethics

The study was performed with permission of the local ethics committee. The head of the board for internal data requests, Dr. U Maeder granted permission to access data from the registry.

### Statistical analysis

The data was analyzed with the statistical software SPSS. Clinical and histological parameters were compared with the Mann–Whitney U or Kruskal–Wallis test for continuous data and with the *χ*^2^ test for categorical variables. *P* < 0.05 was considered statistically significant. Survival curves were drawn according to Kaplan–Meier methods. Cox regression analysis and log rank test were used for multivariate testing [21, 22].

## Results

### Patient characteristics

**Fig. 1:**
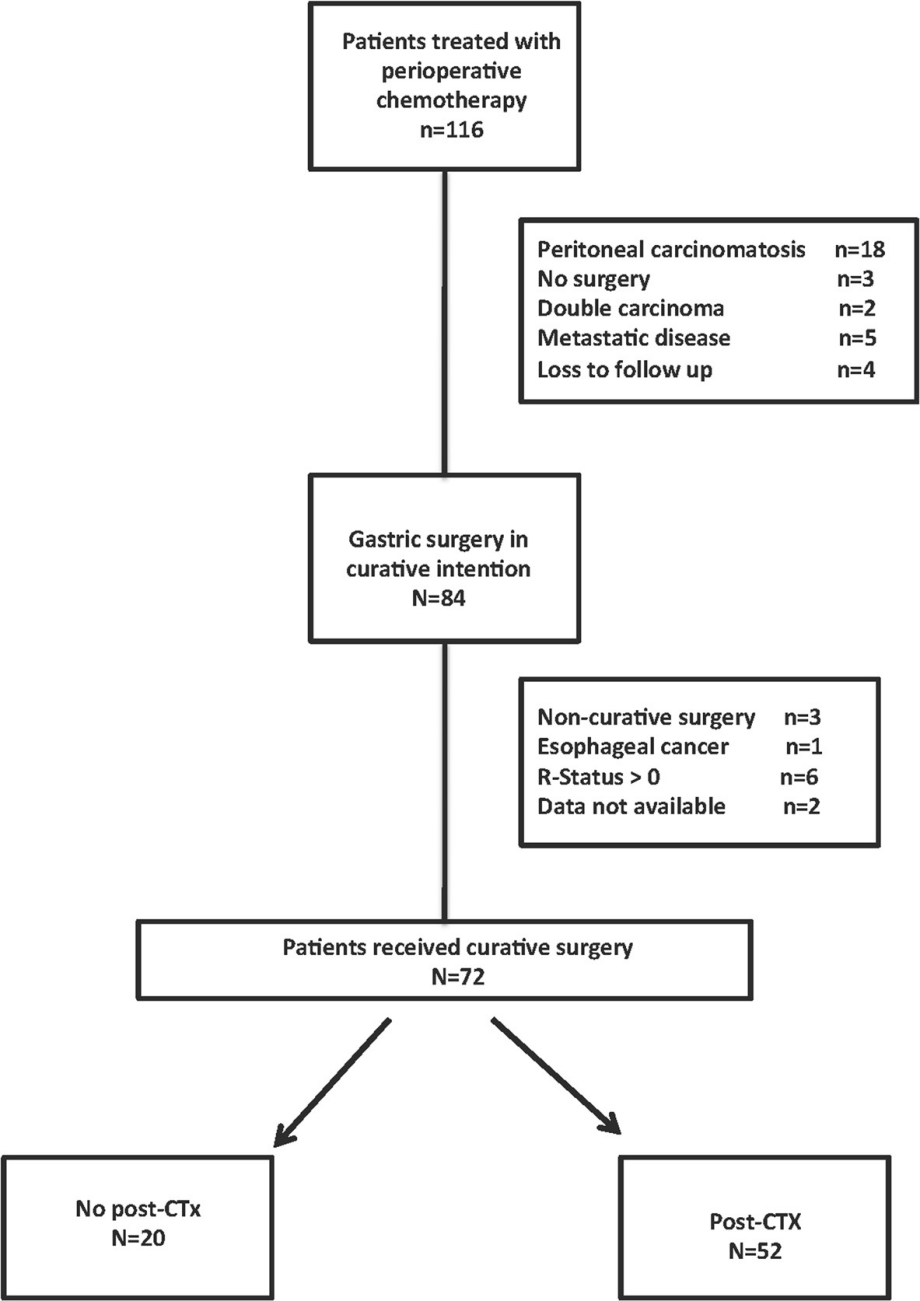
Flow chart of included patients

In total 116 patients, who completed preCTx for locally advanced GC, were identified. The first patient was treated in 2006. 32 patients had to be excluded due to peritoneal carcinomatosis (*n* = 18), liver metastasis (*n* = 5), or a second tumor (*n* = 2). 7 patients refused to undergo surgery. The remaining 84 patients had undergone radical surgical resection (total or subtotal gastrectomy with D2-lymphadenectomy) in curative intention. From this cohort another twelve patients were excluded. The exclusion criteria consisted of the following: R1 resection in nine patients, one esophageal carcinoma and two due to loss of follow up. The remaining cohort consisted of 48 male and 24 female patients (Table 1). The median age at operation was 61.5 years (30.6-83.2) (Fig. 1).

**Table 1:** Clinical and demographic characteristics of 72 patients who underwent curative gastric resection or gastrectomy

Characteristic		Patients total	
		*n* = 72	
		No.	%
Gender	Male	48	66.7
	Female	24	33.3
Age [y]	Median	61.51	
	Range	30.63 – 83.24	
BMI *n* = 62	Median	25.2	
	Range	16.0 – 37.1	
ASA *n* = 71			
	I	5	7.0
	II	42	59.2
	III	24	33.8
Medication			
Cortisone	yes	0	0.0
	no	72	100.0
Immunsup.	yes	2	2.8
	no	70	97.2
HTN	yes	32	44.4
	no	40	55.6
Comorbidities			
DM	no	65	90.3
	NIDDM	7	9.7
	IDDM	0	0.0
CHD	yes	7	9.7
	no	65	90.3
Cirrhosis	yes	0	0.0
	no	72	100.0
COPD	yes	0	0.0
	no	72	100.0
Primary tumor location			
AEG II		22	30.6
AEG III		8	11.1
Corpus		24	33.3
Pylorus		17	23.6
unknown		1	1.4
ypUICC-Stage			
0		8	11.1
I		25	34.7
II		16	22.2
III		23	31.9
ypT-Stage			
0		9	12.5
1		13	18.1
2		19	26.4
3		25	34.7
4		6	8.3
ypN-Stage			
0		44	61.1
1		20	27.8
2		5	6.9
3		3	4.2
ypM-Stage			
0		70	97.2
x		2	2.8

*BMI* body mass index, *ASA* American Society of Anesthesiologists score, *Immunsup* immune suppressing medication, *HTN* blood pressure medication, *DM* diabetes mellitus, *NIDDM* non-insulin depended DM, *IDDM* insulin depended DM, *CHD* coronary heart disease, *COPD* chronic obstructive pulmonary disease, *AEG* adenocarcinoma of the esophageal gastric junction, *UICC* union internationale contre le cancer

The CTx protocols were determined for all patients in a multidisciplinary team conference and changed over time: Between 2006 and 2009 patients received the ECF protocol (epirubicin, cisplatin, 5-fluorouracil) and in 2010 the ECX protocol (epirubicin, cisplatin, capecitabine). Starting in 2011,almost all patients received FLOT (5-fluorouracil, leucovorin, oxaliplatin and docetaxel) or FLO (5-fluorouracil, leucovorin, oxaliplatin). A summary of clinical data is shown in Table 1.

To evaluate the impact of adjuvant chemotherapy in neoadjuvant treated and curatively resected patients with GC we formed two groups, one of which receiving adjuvant chemotherapy and one without. Of 72 curatively resected patients 52 received postCTx (postCTx-group), whereas 20 did not receive postCTX (non-postCTx-group). Reasons for not receiving postCTx in these 20 patients were: 9 patients refused to undergo postCTx and 11 patients were not able to recieve postCTx due to various medical reasons (postoperative death (*n* = 2) or poor general condition (*n* = 9)).

Both groups did not differ regarding gender, BMI, co-morbidities, medication use, tumor depth of invasion (pT-category) or localization. Patients receiving postCTx were slightly younger than patients not receiving postCTx. However, this trend was not statistically significant (*p* = 0.052) (see Table 2).

**Table 2:** Clinical and demographic characteristics of 72 patients that underwent gastric resection or gastrectomy according to application of post-CTx

Characteristic		No adjuvant therapy (*n* = 20)		Adjuvant therapy (*n* = 52)		*p*-value
		No.	%	No.	%	
Gender	Male	12	60.0	36	69.2	0.457
	Female	8	40.0	16	30.8	
Age[y]	Median	64.19		59.89		0.052
	Range	37.13 – 78.77		30.63 – 83.24		
BMI	Median	24.9		25.6		0.817
	Range	18.6 – 35.0		16.0 – 37.1		
ASA						
	I	0	0.0	5	9.8	0.485
	II	15	75.0	27	52.9	
	III	5	25.0	19	37.2	
Medication						
Cortisone	yes	0	0.0	0	0.0	-
	no	20	100.0	52	100.0	
IS	yes	0	0.0	2	3.8	0.374
	no	20	100.0	50	96.2	
HTN	yes	11	55.0	23	44.4	0.953
	no	9	45.0	29	55.8	
Comorbidities						
DM	no	19	95.0	46	88.5	
	NIDDM	1	5.0	6	11.5	0.402
	IDDM	0	0.0	0	0.0	
CHD	yes	1	5.0	6	11.5	0.402
	no	19	95.0	46	88.5	
Cirrhosis Child A		0	0.0	0	0.0	-
	no	20	100	52	100.0	
COPD	yes	0	0.0	0	0.0	-
	no	20	100.0	52	100.0	
Primary tumor location						
AEG II		10	50.0	12	23.1	
AEG III		1	5.0	7	13.5	0.222
Corpus		6	30.0	18	34.6	
Pylorus		3	15.0	14	26.9	
unknown		0	0	1	1.9	
ypUICC-Stage						
0		1	5.0	7	13.5	
I		8	40.0	17	32.7	0.733
II		5	25.0	11	21.2	
III		6	30.0	17	32.7	
ypT-Stage						
0		2	10.0	7	13.5	
1		3	15.0	10	19.2	0.120
2		7	35.0	12	23.1	
3		4	20.0	21	40.4	
4		4	20.0	2	3.8	
ypN-Stage						
0		12	60.0	32	61.5	
1		6	30.0	14	26.9	0.237
2		0	0	5	9.6	
3		2	10.0	1	1.9	
ypM-Stage						
0		20	100	50	96.2	0.374
x		0	0	2	3.8	
Histological type						
diffuse		5	25.0	18	34.6	
intestinal		7	35.0	19	36.5	0.743
mixtype		1	5.0	1	1.9	
unknown		7	35.0	14	26.9	

*CHD* coronary heart disease, *AEG* adenocarcinoma of the esophageal-gastric junction, *HTN* hypertension

### Operation and postoperative complication rate

There was no difference between both groups regarding surgical procedures (subtotal/total/transhiatal gastrectomy) or the operating duration.

When analyzing the complication rates and complication severity grades, patients in the non-postCTx group did not experience more complications or higher complication grades according to the Dindo classification [20]. When analyzing individual postoperative complications, such as acute kidney failure, acute liver failure, pneumonia, re-intubation, tracheotomy, re-operation, anastomotic leakage and time on ICU, a higher occurrence in the postCTx group was observerd, however without statistical significance (see Table 3). By means of multivariate testing (Cox regression), none of the evaluated postoperative factors or the postoperative UICC stage were identified as independent factors for receiving postCTx.

**Table 3:** Operative and postoperative characteristics of 72 patients underwent gastrectomy at the university hospital Wuerzburg according to application of post-CTx

Characteristic		No adjuvant therapy (*n* = 20)		Adjuvant therapy (*n* = 53)		*p*-value
		No.	%	No.	%	
OP-technique	Subtotal	3	15.0	8	15.4	
	Total+Pouch	8	40.0	26	50.0	0.697
	Transhiatal	9	45.0	18	34.6	
OP-Duration *n* = 69	[min]					
	Median	263		261		0.772
	Range	182 – 452		159 – 666		
Time ICU *n* = 72	[d]					
	Median	2		2		0.066
	Range	0 – 24		0 – 17		
Complications						
Endoscopy	No	18	90.0	49	94.2	0.527
	Yes	2	10.0	3	5.8	
Pneumonia	No	18	90.0	50	96.2	0.307
	Yes	2	10.0	2	3.8	
Pulm. embolism	No	20	100	51	98.1	0.532
	Yes	0	0	1	1.9	
Re-Intubation	No	17	85.0	47	90.4	0.515
	Yes	3	15.0	5	9.6	
Tracheotomy	No	19	95.0	52	100	0.104
	Yes	1	5.0	0	0	
AKF	No	19	95.0	52	100	0.104
	Yes	1	5.0	0	0	
ALF	No	19	95.0	52	100	0.104
	Yes	1	5.0	0	0	
Re-Operation	No	18	90.0	47	90.4	0.961
	Yes	2	10.0	5	9.6	
CT-Drainage	No	18	90.0	49	94.2	0.527
	Yes	2	10.0	3	5.8	
Insufficiency	No	17	85.0	49	94.2	0.204
	Yes	3	15.0	3	5.8	
SSI	No	19	95.0	51	98.1	0.477
	Yes	1	5.0	1	1.9	
Wound dehiscence	No	19	95.0	51	98.1	0.477
	Yes	1	5.0	1	1.9	
Clavien-Dindo	II	15	75.0	41	78.8	
	III	4	20.0	11	21.2	0.268
	IV	0	0	0	0	
	V	1	5.0	0	0	

*ICU* Intensive care unit, *SSI* surgical side infection, *AKF* acute kidney failure, *ALF* acute liver failure

### Oncological outcome

**Fig. 2:**
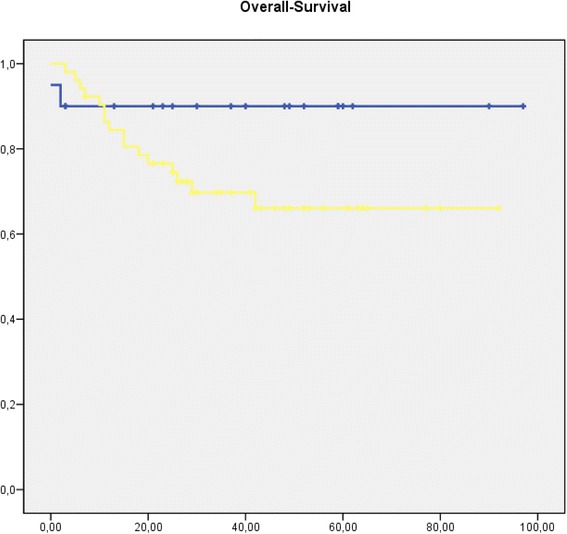
Kaplan-Meier curve showing overall survival from date of operation. [blue: patients without post-CTx (*n* = 20); yellow: patients with post-CTx (*n* = 52) (*p* = 0.1) time in month]

The overall survival of all curative resected patients was 76.3 % after three years follow-up and 75 % after five years of follow-up. Survivals analysis of study groups (postCTx group vs. non-postCTx group) showed a trend towards prolonged survival in the non-postCTx groups, which did not reach statistical significance (*p* = 0.101). (Fig. 2)

**Fig. 3:**
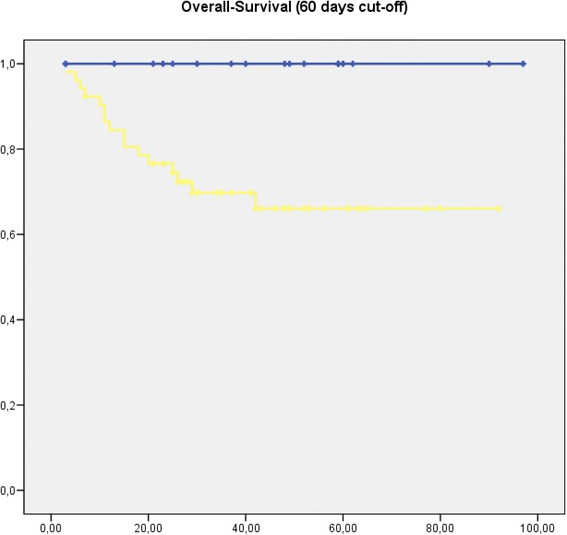
Kaplan-Meier curve showing overall survival from 60 days postoperative. [blue: patients without post-CTx (*n* = 18); yellow: patients with post- CTx (*n* = 52) (*p* = 0.038) time in month]

Two patients died due to postoperative complications. Per protocol analysis after exclusion of these two patients revealed significantly worse long-term survival at 3 years (postCTx-group 71.2 % vs. non-postCTx-group 100 %), as well as 5 years (postCTx-group 69.2 % vs. non-postCTX-group 100 %; *p* = 0.038) for patients treated with postCTx. (Fig. 3)

**Fig. 4:**
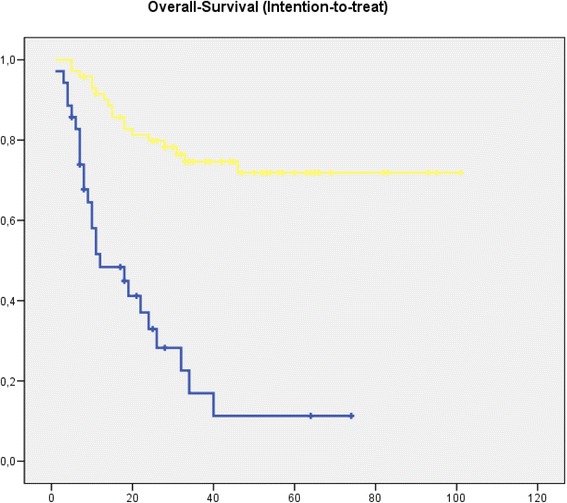
Kaplan-Meier curve showing overall survival from date of diagnosis. All patients received neoadjuvant therapy. [blue: patients without curative resection (*n* = 35); yellow: patients with curative resection (*n* = 72) (*p* < 0.01) time in month]

When performing an intention to treat analysis with all patients, who underwent preCTX, and stratifying for curative resection, patients, who could not be resected, demonstrated a worse outcome with a 3-year survival of 31.4 % compared to 76.3 % after curative resection independent of postCTx (*p* < 0.01). (Fig. 4)

## Discussion

Currently, the use of perioperative CTx in addition to radical surgical resection (D2-gastrectomy) is the accepted standard therapy for advanced gastric cancer, as laid down by experts and in evidence-based guidelines [23]. These perioperative CTx protocols consist of preCTx as well as postCTx. However, the role of the postoperative component of this strategy (postCTx) is not entirely clear yet.

The evidence, on which perioperative CTx has been established as standard therapy for the treatment of locally advanced gastric cancer, still underlies controversial debate.

It is well known that only a subset of patients receive postCTx due to a variety of reasons. In our study 45 % refused the CTx and 55 % did not receive post CTx due to various medical reasons.

In Europe, perioperative chemotherapy for high-risk gastric cancer is the standard therapy for high-risk gastric cancer based primarily on the results of three large, randomized trials: the UK-MAGIC Trial by Cunningham [17], the French FNCLCC/FFCD phase III trial [18] and the European Organisation for Research and Treatment of Cancer Randomized Trial 40954 [19]. In the MAGIC trial patients undergoing perioperative chemotherapy with ECF (epirubicin, cisplatin and fluorouracil) had a significant higher five-year survival rate (36 % compared to 23 % without chemotherapy) without showing differences in the postoperative complication rate. Similarly, the French FNCLCC/FFCD phase III trial showed a significant improved 5-year overall survival rate of 38 % compared to 24 % for patients receiving perioperative chemotherapy with cisplatin and fluoruracil. So far the point in time of additional chemotherapy (pre- / peri- / postoperative) has not been addressed sufficiently as starting (50-66 %) and completion (23-42 %) rates of postoperative chemotherapy is low. Although the results from the MAGIC trial constitute the basis for our current recommendations of perioperative CTx for gastric cancer, they have been severely criticized for several reasons [17]. Points of criticism have been, for example the low quality of surgery. Only a minority of patients received radical D2-gastrectomy, which is regarded as the standard for adequate radical resection. Furthermore, the trail included locally limited tumors (T1/ T2 categories), which only require radical surgery, but no CTx. With regard to this paper’s topic, the most important point of criticism of the MAGIC trail is, that only a minority of patients (<50 %) received postCTx, thereby violating the protocol of perioperative CTx. In conclusion, the impact of postoperative chemotherapy in patients with completed preoperative chemotherapy remains unclear.

In this retrospective analysis of a highly selected population of patients with completed neoadjuvant chemotherapy and curative surgery we found significant higher survival rates in patients without postoperative chemotherapy in comparison to those, who completed the perioperative therapy. This is, in several ways, surprising.

A. the observed overall survival independant of the postoperative chemotherapy is better than reported so far.
B. With nearly 90 % three-year survival the patient group without postoperative chemotherapy displays a long-term survival that is comparable to patients with early stage GC [24].
C. The patients without postoperative chemotherapy did not have statistically more or more severe complications.

The exact reasons for the worse outcome in the group receiving postCTx are not known. It can be speculated that there is a negative selection bias for patients recieving postCTx, but this seems to be unlikely as there are no differences in the pathological UICC stage. Also, the anatomical distribution is different with more proximal located tumors in the non-postCTX group, without showing a statistical significant difference. Overall, a selection bias for the administration of postoperative chemotherapy cannot be ruled out in this analysis. Especially not addressed factors, such as the response rate to preCTX, could influence the results.

In general, perioperative chemotherapy has several advantages compared to postoperative chemotherapy. It has a higher tolerability prior to a potentially debilitating surgical procedure. Preoperative chemotherapy could lead to down staging of the tumor and improved R0 resection rates. There is an early systemic treatment of micro metastatic disease and a detection of tumors with a worse biological phenotype which lead to progression under chemotherapy. Taking in account the comparable postoperative complication rate with or without postCTx and our discussed data, it can be argued that preoperative chemotherapy should be the only admitted chemotherapy and should perhaps be extended [25].

An often-discussed potential disadvantage of preCTx is the delay of a potentially curative surgery due to neoadjuvant chemotherapy toxicity. However several points argue against this: A) only local advanced cancers should be treated with neoadjuvant chemotherapy. Patients with small tumors with a good long-term survival undergo curative surgery directly rather than receiving preoperative chemotherapy [23]. B) Tumors qualifing for neoadjuvant treatment have a very bad prognosis with surgery alone and only the administration of any kind of chemotherapy can enhance the prognosis. C) Both the MAGIC and French trial showed that 92–96 % of patients who received preoperative chemotherapy underwent surgery.

Our study has several limitations that will be addressed in the following. Firstly, since this is an uncontrolled, retrospective study, patient selection bias cannot be ruled out, as clinicians are more likely to recommend post operative therapy to younger, healthier patients, who would be expected to tolerate chemotherapy better than older patients of poorer performance status. In our cohort, patients with adjuvant chemotherapy were younger and thereby may have had worse tumor biology. However, ASA score, co-morbidities and UICC stage did not differ between the postCTx- and non-postCTX-group. Secondly, patients who died during the first 60 days postoperative were included as well. They, by history, cannot undergo adjuvant chemotherapy thereby reducing the survival rate in the “no adjuvant therapy” group. But when analyzing the survival rate without these patients, the survival benefit lies in the “no adjuvant therapy” group. Thirdly, the chemotherapy protocols have changed over the study period and cannot be compared directly to one another. Fourthly, the reason for not receiving post-CTX is not associated with acute postoperative complication rate which has so far been supposed as negative predictive factor for survival. Another independent factor for the application of adjuvant CTx is the performance and nutrition status at the 3 month check-up. This has not been archived in our system and thus cannot be evaluated.

## Conclusion

The results presented, while speculative, question the value of post-CTx in gastric cancer patients treated with preoperative chemotherapy and curative surgery. It is worthwhile to consider prospective, randomized trials of perioperative CTx versus a purely neoadjuvant approach.

## Abbreviations

GG: gastric cancer
TX: chemotherapy
